# Metabolomic biomarkers in liquid biopsy: accurate cancer diagnosis and prognosis monitoring

**DOI:** 10.3389/fonc.2024.1331215

**Published:** 2024-02-07

**Authors:** Wenqian Wang, Shanshan Zhen, Yu Ping, Liping Wang, Yi Zhang

**Affiliations:** ^1^ Biotherapy Center, The First Affiliated Hospital of Zhengzhou University, Zhengzhou, Henan, China; ^2^ Department of Oncology, The First Affiliated Hospital of Zhengzhou University, Zhengzhou, Henan, China; ^3^ Key Laboratory for Tumor Immunology and Biotherapy of Henan Province, Zhengzhou, Henan, China; ^4^ School of Life Sciences, Zhengzhou University, Zhengzhou, Henan, China; ^5^ State Key Laboratory of Esophageal Cancer Prevention and Treatment, Zhengzhou University, Zhengzhou, Henan, China

**Keywords:** liquid biopsy, metabolomics, cancer biomarkers, diagnosis, prognosis

## Abstract

Liquid biopsy, a novel detection method, has recently become an active research area in clinical cancer owing to its unique advantages. Studies on circulating free DNA, circulating tumor cells, and exosomes obtained by liquid biopsy have shown great advances and they have entered clinical practice as new cancer biomarkers. The metabolism of the body is dynamic as cancer originates and progresses. Metabolic abnormalities caused by cancer can be detected in the blood, sputum, urine, and other biological fluids via systemic or local circulation. A considerable number of recent studies have focused on the roles of metabolic molecules in cancer. The purpose of this review is to provide an overview of metabolic markers from various biological fluids in the latest clinical studies, which may contribute to cancer screening and diagnosis, differentiation of cancer typing, grading and staging, and prediction of therapeutic response and prognosis.

## 1 Introduction

The occurrence and development of cancer is an ever-evolving process that challenges tissue biopsy as the current gold standard for cancer diagnosis (1, 2). Compared to tissue biopsy, liquid biopsy has the advantages of non-invasiveness, high sensitivity, and repeatable sampling, which makes it a promising approach to overcome cancer heterogeneity and realize dynamic monitoring of cancer (3, 4). The exploration of circulating free DNA (5), circulating tumor cells (6), and exosomes (7) is moving from basic research to clinical applications. The initial success of liquid biopsy has also accelerated extensive research into various forms of potential biomarkers (3, 8), such as metabolites, tumor-educated platelets, immune cell components, and proteins.

Metabolic competition poses new challenges for the growth and survival of cancer cells. For one thing, cancer cells have to alter their metabolic fluxes to gain a competitive advantage in the tumor microenvironment (TME) and meet their biosynthetic and energetic requirements. For another, the abnormal accumulation of metabolites in the TME makes it necessary for cancer cells to metabolically adapt to protect themselves from harm. As a result, there is a growing appreciation for alterations in metabolites and their metabolic pathways in cancer. Metabolomics is the scientific discipline that studies the dynamics of metabolites and their mechanisms in organisms under physiological and pathological conditions (9). As a favorable complement to genomics, transcriptomics, and proteomics, the initial value of metabolomics is as a tool for identifying disease biomarkers, as changes in metabolites are regulated by upstream signaling molecules (10). Concurrently, changes in the “quality” and “quantity” of metabolic molecules will in turn affect the replication and expression of genes. Certain metabolites tend to change earlier than genes and proteins, which can reflect fluctuations in the body state in a more timely and effective manner (11). In a sense, metabolomics is also making itself noticed as a “commander.” Metabolomics can be classified into non-targeted and targeted metabolomics based on the study’s purpose. The former can provide a wide range of differential metabolite profiles, whereas the latter focuses on the absolute quantification of a specific set of metabolites (11). The two most frequently used analytical techniques in metabolomics are nuclear magnetic resonance (NMR) and mass spectrometry (MS) (12, 13). Combined MS technologies, such as liquid chromatography-mass spectrometry (LC-MS) and gas chromatography-mass spectrometry (GC-MS), are increasingly used (11, 13).

**Figure 1:**
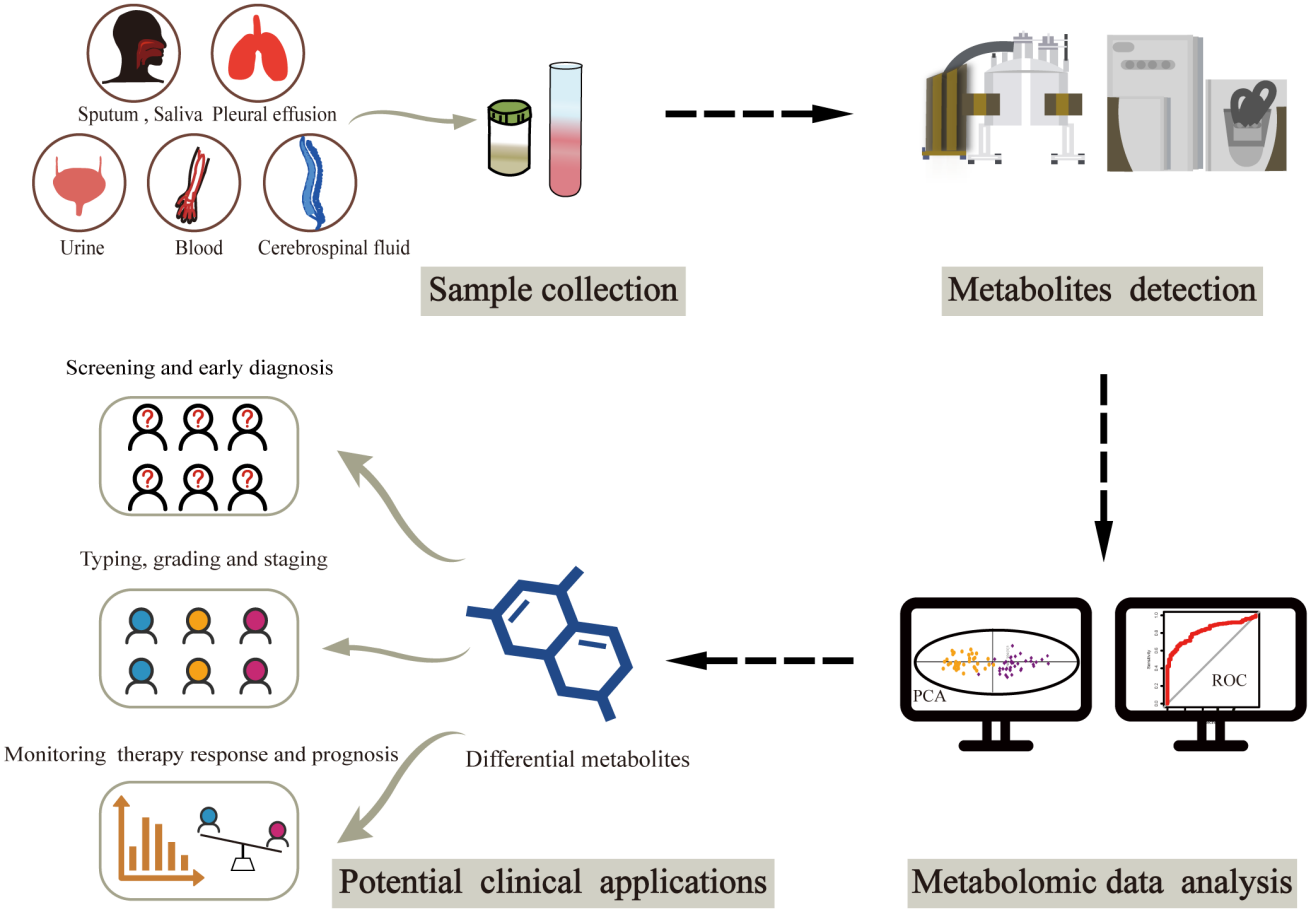
Workflow of metabolomics-based liquid biopsy in cancer. After collecting samples from various biofluids, metabolites can be detected by NMR and/or MS. Differential metabolites are then obtained through metabolomic data analysis, which can be used as biomarkers for accurate cancer diagnosis and classification as well as prognosis prediction. NMR, nuclear magnetic resonance; MS, mass spectrometry.

The clinical applications of metabolomics have been largely restricted to metabolic imaging (14, 15). However, the recent emergence of liquid biopsy has sparked interest in the potential role of circulating metabolic molecules, opening new avenues for cancer biomarker discovery (**Figure 1**). There is an ongoing search for biomarkers in circulating body fluids, mostly from blood and urine, using metabolomics. Owing to the pathophysiological characteristics of different cancers, options for biological sample types are increasingly diverse. A small number of studies have focused on metabolites from other biological samples such as sputum, cerebrospinal fluid and pleural effusion. Herein, we summarize the latest metabolic findings based on liquid biopsy in cancer population studies, intending to identify potential metabolic markers that could aid in clinical cancer diagnosis, classification, and treatment. Finally, we discuss the current limitations and future directions in the field of liquid biopsy-based cancer metabolomic biomarker research.

## 2 Metabolomic biomarkers for cancer screening and diagnosis

The early clinical symptoms of cancer are hidden, and most patients are in the middle and advanced stages once diagnosed, resulting in the loss of opportunities for radical surgery. Therefore, the key to prolonging the survival of patients with cancer is to identify high-risk groups and achieve an early diagnosis. In the search for potential metabolic markers, numerous metabolomic studies have recently compared the metabolic differences in biofluids between patients with cancer and healthy individuals or patients with benign lesions. In this section, we screen differential metabolites mentioned in at least three previous studies (**Table 1**) and outline how these metabolites change in patients with cancer.

**Table 1:** Summary of biofluid metabolomic biomarkers for screening and early diagnosis in cancer.

Metabolite	Sample	Diseases	Refs
Glucose metabolism			
Glucose	Serum/plasma	↑: Kidney cancer	(16)
		↓: Diffuse large B-cell lymphoma	(17)
	Urine	↓: Prostate cancer	(18)
Pyruvate	Serum/plasma	↑: Esophageal cancer, non-small cell lung cancer	(19, 20)
	Urine	↑: Prostate cancer	(18)
Lactic acid	Serum/plasma	↑: Osteosarcoma	(21)
		↓: Kidney cancer	(16)
	Urine	↑: Prostate cancer, renal cell carcinoma	(22, 23)
Myoinositol	Serum/plasma	↑: Prostate cancer	(24)
		↓: Kidney cancer, lung cancer	(16, 25)
	Urine	↑: Renal cell carcinoma, pancreatic cancer	(22, 26)
Amino acid metabolism			
Glutamic acid	Serum/plasma	↑: Hepatocellular carcinoma, osteosarcoma, pancreatic cancer, small cell lung cancer, kidney cancer, esophageal squamous cell carcinoma	(21, 27–31)
		↓: Pancreatic cancer, prostate cancer, colorectal cancer, epithelial ovarian cancer	(23, 32–34)
	Urine	↑: Renal cell carcinoma,	(22)
Glutamine	Serum/plasma	↑: Esophageal squamous cell carcinoma, prostate cancer, epithelial ovarian cancer	(23, 31, 33)
		↓: Lung cancer, pancreatic cancer, pan-cancer	(32, 35, 36)
Leucine	Serum/plasma	↓: Kidney cancer, small cell lung cancer	(16, 29)
	Urine	↑: Prostate cancer	(18, 37)
	Saliva	↓: Oral squamous cell carcinoma	(38)
Isoleucine	Serum/plasma	↓: Pancreatic ductal adenocarcinoma	(39)
	Urine	↑: Prostate cancer, gastric cancer	(37, 40)
Valine	Urine	↑: Prostate cancer	(18)
	Saliva	↓: Lung cancer, thyroid cancer	(41, 42)
Tryptophan	Serum/plasma	↑: Non-small cell lung cancer, colorectal cancer	(19, 43, 44)
		↓: Pancreatic cancer, bladder cancer	(32, 45)
	Saliva	↓: Lung cancer	(46)
Glycine	Serum/plasma	↓: Kidney cancer, endometrial cancer, esophageal cancer	(16, 47, 48)
	Urine	↓: Esophageal cancer	(48)
Serine	Serum/plasma	↑: Lung cancer, prostate cancer	(24, 35)
		↓: Endometrial cancer	(47)
	Urine	↑: Gastric cancer	(40)
	Saliva	↓: Lung cancer	(41)
Methionine	Serum/plasma	↑: Epithelial ovarian cancer, non-small cell lung cancer	(19, 33)
		↓: Small cell lung cancer	(29)
	Saliva	↑: Oral squamous cell carcinoma	(38)
Cysteine	Serum/plasma	↑: Lung adenocarcinoma, gastric cancer, oral squamous cell carcinoma	(49–51)
Proline	Serum/plasma	↑: Lung cancer, pan-cancer	(36, 52)
		↓: Hepatocellular carcinoma, pancreatic cancer	(27, 53)
	Saliva	↓: Thyroid cancer, lung cancer	(41, 42)
Phenylalanine	Serum/plasma	↑: Non-small cell lung cancer, lung cancer	(54–56)
		↓: Pancreatic cancer	(32)
Phenylalanine	Saliva	↓: Thyroid cancer	(42)
Tyrosine	Serum/plasma	↑: Hepatocellular carcinoma, non-small cell lung cancer	(19, 57)
	Saliva	↓: Lung cancer	(46)
Nucleotide metabolism			
Hypoxanthine	Serum/plasma	↑: Esophageal squamous cell carcinoma, breast cancer	(31, 58)
		↓: Esophageal squamous cell carcinoma	(59)
Inosine	Plasma	↓: Pancreatic cancer, esophageal squamous cell carcinoma	(59, 60)
	Saliva	↑: Oral squamous cell carcinoma	(38)
Uracil	Serum	↑: Lung adenocarcinoma	(49)
		↓: Breast cancer	(61)
	Saliva	↑: Oral squamous cell carcinoma, lung cancer	(38, 41)
Lipid metabolism			
Palmitic acid	Serum/plasma	↑: Non-small cell lung cancer, pancreatic cancer	(19, 53)
		↓: Lung cancer, gastric cancer	(62, 63)
Linoleic acid	Serum/plasma	↓: Esophageal squamous cell carcinoma, colorectal cancer, gastric cancer	(31, 44, 63)
Phosphatidylcholines	Serum/plasma	↑: Hepatocellular carcinoma	(64)
		↓: Lung cancer, pancreatic cancer, hepatocellular carcinoma	(28, 64, 65)
	Pleural effusion	↓: Lung cancer	(66)
Lysophosphatidylcholines	Serum/plasma	↑: Colorectal cancer	(44)
		↓: Pancreatic cancer, gastric cancers, renal cell carcinoma, colorectal cancer, esophageal squamous cell carcinoma, lung cancer	(35, 54, 59, 63, 67–69)
	Pleural effusion	↓: Lung cancer	(66)
Phosphatidylethanolamines	Serum/plasma	↑: Pancreatic cancer	(28, 70)
	Pleural effusion	↓: Lung cancer	(66)
Lysophosphatidylethanolamines	Serum/plasma	↑: Esophageal squamous cell carcinoma	(59)
		↓: Pancreatic ductal adenocarcinoma, colorectal cancer	(69, 70)
Sphingomyelins	Serum/plasma	↑: Ovarian cancer, papillary thyroid cancer, pancreatic ductal adenocarcinoma, endometrial cancer	(47, 70–72)
		↓: Laryngeal cancer,	(73)
Ceramides	Serum/plasma	↑: Ovarian cancer	(71)
		↓: Pancreatic cancer	(67, 70)
Glycocholic acid	Serum/plasma	↑: Colon cancer, hepatocellular carcinoma, pancreatic cancer	(57, 60, 74)
Estradiol	Serum	↑: Breast cancer	(75)
	Urine	↑: Breast cancer	(76, 77)
Others			
Hippuric acid	Urine	↓: Prostate cancer, renal cell carcinoma, bladder cancer	(23, 78, 79)

Up arrow indicates increase, down arrow indicates decrease.

### 2.1 Glucose metabolism

#### 2.1.1 Glucose

Glucose is a vital nutrient that tumor cells compete whose uptake depends largely on glucose transporters (GLUTs) on the cell membranes. There are four classical GLUTs in the human body, among which GLUT1 and GLUT3 have high glucose affinities and are upregulated in multiple tumor types (80). Significant differences in circulating glucose levels between cancer-free controls and patients with cancer have been reported in recent studies (16–18). Using GC-MS techniques, two studies detected relatively low levels of glucose in the plasma of patients with diffuse large B-cell lymphoma and in urine from patients with prostate cancer (17, 18), which may potentially result from increased glucose uptake by tumor cells from the peripheral circulation. In contrast, Nizioł et al. observed higher glucose concentrations in patients with renal cancer than in healthy controls using MRI spectroscopy (16), which appears to contradict the theory of active glucose metabolism in tumor tissues. Considering the characteristics of renal blood flow regulation, it is more likely a stress glucose elevation caused by sympathetic excitation during renal ischemia (81). Taken together, these metabolomic findings suggest that changes in glucose levels in biological fluids may help in the early identification of patients with cancer.

#### 2.1.2 Pyruvate

Pyruvate synthesis relies primarily on the glycolytic pathway, and the conversion of glucose to glucose-6-phosphate catalyzed by hexokinase is the first rate-limiting step. KRAS mutation has been reported to directly regulate the enzymatic activity of hexokinase 1, thereby increasing glucose utilization by tumor cells (82). Overexpression of phosphofructokinases and pyruvate kinases in tumor tissues has also been confirmed to be involved in tumor growth and invasion (83, 84). Several studies have consistently found that patients with cancer show relatively high levels of pyruvate in the blood and urine. In addition to decreased glucose levels, Lima et al. observed a marked increase in urinary pyruvate levels in patients with prostate cancer (18). In another study, by combining differential metabolites obtained by MS with a metabolic network based on Kyoto Encyclopedia of Genes and Genomes database, Guo et al. constructed a metabolite panel (including pyruvate) specifically for non-small cell lung cancer (NSCLC) with a diagnostic accuracy of almost 98% (19). Subsequently, by applying the NMR technique, Ye et al. proposed that the combination of pyruvate and acetate presented significantly superior diagnostic performance compared to carbohydrate antigen 199 (CA199) and carcinoembryonic antigen (CEA) and could serve as a candidate marker for the diagnosis of esophageal cancer (20).

#### 2.1.3 Lactic acid

Aerobic glycolysis, known as the “Warburg effect,” is a classic example of metabolic adaptation in tumor cells; even under aerobic conditions, glucose in tumor cells is not completely oxidized but is catabolized to lactic acid, which provides a large carbon source for tumor cells while rapidly generating adenosine triphosphate (85, 86). Lactic acid transport relies on monocarboxylic acid transporters (MCTs), in which abnormal expression of MCT1 and MCT4 in different tumor types affects intracellular and extracellular lactic acid homeostasis, thereby participating in distant metastasis and recurrence (87, 88). To explore the serum metabolic profile of osteosarcoma, Lv et al. applied non-targeted metabolomics techniques to identify the metabolites that differ between patients with cancer and healthy individuals. The area under the curve (AUC) of lactic acid was 0.97 according to receiver operating characteristic (ROC) analysis (21). Using benign lesions as controls, Sato et al. demonstrated that urine lactic acid levels were significantly elevated in patients with renal cell carcinoma, which was consistent with their previous metabolomic analysis based on tumor tissue samples (22). Conversely, another metabolomic study of kidney cancer reported lower serum lactic acid levels in patients with cancer (16). Although the phenomenon discussed in the above two studies requires further confirmation, variations in inclusion criteria for controls across studies may influence the alteration of lactate levels in blood and urine.

#### 2.1.4 Myoinositol

Inositol is a class of cyclic sugar alcohols with nine isomer forms that are widely distributed throughout the body, among which myoinositol is the most stable. In addition to dietary intake, myoinositol can be synthesized *in vivo* by inositol-3-phosphate synthase 1 (ISYNA1) and inositol monophosphatase catalysis (89). Zhou and colleagues found a positive correlation between ISYNA1 expression and the survival of patients with pancreatic cancer, and observed increased invasiveness in ISYNA1-silenced pancreatic cancer cells (90). However, ISYNA1 plays the opposite role in bladder cancer, and its knockdown significantly promotes apoptosis of bladder cancer cells (91). Three studies reported increased myoinositol levels in prostate cancer (24), renal cell carcinoma (22) and pancreatic cancer (26), whereas two studies reported decreased myoinositol levels in kidney cancer (16) and lung cancer (25). These studies have confirmed the effectiveness of metabolic panels, including myoinositol, in the early diagnosis of cancer.

### 2.2 Amino acid metabolism

#### 2.2.1 Glutamine and glutamic acid

Glutamine is a crucial nutrient for sustaining cell proliferation and survival, especially in tumor cells. Solute carrier family 1 member 5 is a key protein responsible for glutamine transport, and its upregulation in tumor tissues indicates increased glutamine uptake (92). Glutamate is the breakdown product of glutamine, catalyzed by glutaminase, which can be further converted to α-ketoglutarate (α-KG) by glutamate dehydrogenase or aminotransferase. On the one hand, α-KG entering the tricarboxylic acid (TCA) cycle replenishes the intermediate products of aerobic oxidation and promotes the production of adenosine triphosphate. On the other hand, α-KG can also further enter the fatty acid synthesis pathway by converting to citric acid, which is more pronounced in the hypoxic TME (93, 94). In addition, it has been demonstrated that regulatory activation of glutaminases promotes tumor progression by producing more glutathione to assist tumor cells in resisting oxidative stress (95). Numerous studies have reported changes in glutamine and glutamate levels in the blood and urine of cancer patients, and suggested that these two metabolites could be used as potential metabolic markers to effectively distinguish cancer patients from non-cancerous individuals. Seven studies found higher levels of glutamine (21, 22, 27–31) and three studies observed lower glutamine levels in patients with cancer (32, 35, 36). These findings may be attributed to enhanced glutamine catabolism, which produces more glutamate. It is worth mentioning that in a study on esophageal squamous cell carcinoma, the authors found serum glutamine and glutamate levels were significantly higher in cancer patients compared to healthy controls (31). While only glutamate showed a statistically significant difference when patients with dysplasia were used as controls, this may indicate that glutamate and glutamine metabolism dynamically change during cancer progression. However, in two other studies based on serum metabolomics, an increase in glutamine and a decrease in glutamate levels were observed in ovarian and prostate cancers, respectively (23, 33). This implies that reproductive system tumors are likely to be less dependent on glutamine metabolism. Similarly, one study found significantly lower blood glutamate levels in patients with colon cancer than in those with colon adenomas (34), although more work is needed to verify the glutamate differences between patients with colon cancer and healthy individuals.

#### 2.2.2 Branched-chain amino acids (BCAAs)

BCAAs refer to three non-essential amino acids, namely leucine, isoleucine, and valine, which are derived from exogenous food intake and endogenous tissue protein breakdown. The upregulation of L-type amino acid transporter 1, induced by hypoxia-inducible factor in the TME can promote more BCAAs into cells (96). The catabolism of BCAAs mediated by amino acid branched-chain transaminases is abnormally active in pancreatic malignancies (97, 98). Based on serum metabolomics, two studies observed reduced leucine levels in renal tumors and small cell lung cancer (SCLC), respectively (16, 29). Consistent with these findings, patients with pancreatic ductal adenocarcinoma also had reduced levels of two isomers of leucine: isoleucine and ortholeucine (39). Decreased levels of BCAAs have also been detected in saliva samples from patients with cancer, including oral squamous cell carcinoma, lung cancer and thyroid cancer (38, 41, 42), implying vigorous catabolism of BCAAs in cancer cells to meet the energy requirements of biological macromolecules. However, urinary metabolomics showed the opposite trend. Two studies found that patients with prostate cancer have higher levels of BCAAs in their urine than controls (18, 37). Based on MS and binary logistic regression analysis methods, Huang’s team observed a decline in urinary isoleucine in patients with gastric cancer and constructed an early diagnostic model by combining differential metabolites with age (40). It is worth noting that considering the effect of age on human metabolism, taking age into account somewhat improved the reliability of the diagnostic model in this study.

#### 2.2.3 Amino acids in one-carbon metabolism

One-carbon metabolism not only provides raw materials for nucleotide synthesis and methylation reactions in tumor cells but also participates in maintaining cellular redox homeostasis, which is an important component of metabolic reprogramming in tumor cells (99).

Serine and glycine are the main sources of one-carbon units for tumor cells. In the serine-restricted TME, tumor cells can regulate the expression of phosphoglycerate dehydrogenase, allowing more 3-phosphoglycerate to enter the serine synthesis pathway (100). Published studies regarding the variation of serine content in biofluids are inconsistent. Yang et al. proposed a diagnostic model of metabolites containing serine for early-stage lung cancer with an AUC of more than 95% (35). Similarly, an MS study on prostate cancer demonstrated that differential serum metabolites (L-serine, myoinositol and decanoic acid) performed better than prostate specific antigen in distinguishing patients with prostate cancer from benign prostate hyperplasia (24). Both studies confirmed higher serine levels in patients with cancer whereas serum glycine and serine levels were inversely correlated with the risk of endometrial cancer in an observational study (47). Further studies are needed to determine whether these two metabolites are directly involved in the pathological process of endometrial cancer. Moreover, there is a notable disparity in serine levels in urine and sputum samples from individuals with cancer compared to those without cancer (40, 41). The conversion of serine to glycine requires catalysis by serine-hydroxymethyltransferase, an important target for the regulation of lung cancer metastasis (101). Several studies have consistently found relatively low levels of glycine in the circulating body fluids of cancer patients (41, 47, 48).

Tryptophan catabolism can also produce one-carbon units. Abnormalities in tryptophan metabolism in tumor cells have been extensively studied and metabolites involved in tryptophan catabolism, such as kynurenine, have emerged as targets for anti-tumor therapy (102). Alterations in tryptophan levels in the biological fluids of lung cancer patients have been observed in three studies, two of which found that patients with NSCLC had elevated blood tryptophan levels compared to healthy controls (19, 43). In contrast, a study based on sputum metabolomics observed the opposite trend in patients with lung cancer (46), and the difference in the distribution of tryptophan in blood and sputum may be the result of increased tryptophan consumption by tumor cells. In addition to lung cancer, there are significant changes in circulating blood tryptophan levels in colorectal, pancreatic, and bladder cancer (32, 44, 45).

Methionine can provide methyl groups for the synthesis of various bioactive substances, such as carnitine, choline, and creatine, through transmethylation reactions; therefore, there is intense competition for methionine in the TME. High expression of methionine transporters in tumor cells results in a stronger methionine uptake capacity than that of CD8+ T cells, thereby limiting the anti-tumor function and survival of CD8+ T cells (103). Changes in blood methionine levels have shown significance in the diagnosis of lung cancer, although they have an opposite trend in SCLC and NSCLC (19, 29), suggesting metabolic heterogeneity between the different pathological types of lung cancer. Besides, increased methionine and spermidine levels were observed in patients with oral squamous cell carcinoma in a salivary metabolomics study (38).

The transsulfuration pathway refers to the synthesis progress of cystine by serine and homocysteine under the catalysis of cystathionine β-synthase and cystathionine-γ-lyase, which is the main source of cystine synthesis *in vivo*. Cystathionine-γ-lyase is the key enzyme in this process and its high expression in tumor tissues has emerged as a promising target for cancer therapy (104, 105). Patients with lung adenocarcinoma (49), gastric cancer (50), and oral squamous cell carcinoma (51) show elevated cysteine levels in their blood, which also reflects the disorder of cysteine metabolism in the tumor state, demonstrating that cysteine can be used as a biomarker for cancer early detection.

#### 2.2.4 Proline

As a non-essential amino acid, in addition to exogenous food intake, proline comes from the *de novo* synthesis pathway and collagen degradation (106). The conversion between pyrroline-5-carboxylate and proline is a reversible reaction catalyzed by pyrroline-5-carboxylate reductase and proline dehydrogenase; the former has been consistently recognized as a key oncogenic factor that promotes tumor proliferation and maintains redox homeostasis (107), while proline dehydrogenase plays a dual role in tumors by regulating apoptosis and autophagy in tumor cells (108). There are also conflicting results regarding changes in blood proline levels in patients with tumors, and two studies reported elevated proline levels compared to controls (36, 52), which may be associated with the high expression of pyrroline-5-carboxylate reductase in tumors. However, a multicenter study found that the serum proline level in patients with pancreatic cancer was lower than that in healthy controls and benign lesion groups and proposed a metabolic panel (proline, creatine and palmitic acid) that showed good sensitivity and specificity in the early diagnosis of pancreatic cancer (53). Sputum proline levels have also been reported, with two studies showing reduced proline levels in patients with thyroid and lung cancer (41, 42).

#### 2.2.5 Phenylalanine and tyrosine

The conversion of phenylalanine to tyrosine, catalyzed by phenylalanine hydroxylase is the main metabolic pathway of phenylalanine in the human body. In addition to being the main source of catecholamine neurotransmitters, tyrosine is also converted to janosonic acid and acetoacetic acid, catalyzed by tyrosine aminotransferase, and then enters the TCA cycle and lipid metabolism pathway, respectively. Cang et al. found elevated plasma levels of phenylalanine in NSCLC and suggested that this may be due to abnormal inflammation and immune responses in tumors, which impair the function of phenylalanine hydroxylase (54). Similarly, two recent studies have reported increased levels of phenylalanine in the blood of lung cancer patients (55, 56). In another metabolomic study, patients with lung cancer showed increased sputum levels of tyrosine compared to those with benign lung lesions, and a metabolic panel combining tyrosine with diethanolamine, cytosine, and lysine was proposed for the early diagnosis of lung cancer (46). Changes in phenylalanine and tyrosine levels in other cancers have also been reported (32, 42, 57). One of these observational studies demonstrated a good positive correlation between tyrosine content and hepatocellular carcinoma based on serum metabolomics (57).

### 2.3 Nucleotide metabolism

#### 2.3.1 Inosine and hypoxanthine

Inosine and hypoxanthine are important intermediate products of purine metabolism that are produced sequentially by the decomposition of hypoxanthine nucleotides. Hypoxanthine can enter the salvage synthesis pathway to participate in nucleotide synthesis again or be further oxidized by xanthine oxidase to form uric acid and excreted from the body. To realize the early diagnosis of esophageal squamous cell carcinoma, Zhu et al. conducted a comparative analysis of serum metabolites in 140 patients with cancer and 170 healthy controls and established a panel consisting of eight metabolites among which inosine and hypoxanthine levels were relatively low in the cancer cohort compared to those in the healthy cohort (59). In contrast, using abnormal esophageal squamous hyperplasia as a control, Zhang et al. obtained a completely different panel containing increased hypoxanthine, l-glutamate, l-aspartate and decreased 2-ketoisocaproic acid (31). Altered plasma levels of inosine and hypoxanthine were also found in patients with pancreatic and breast cancer in two other MS studies (58, 60).

#### 2.3.2 Uracil

Uracil is derived from the catabolism of pyrimidine nucleotides in the liver, which can be further catabolized to β-alanine, a key mediator of metabolic crosstalk between tumor and stromal cells (109). In addition to being excreted in urine, more importantly, β-alanine can be broken down into acetyl coenzyme A, providing energy and raw materials for cellular biosynthesis. There is inconclusive evidence on the changes in uracil levels among cancer patients, as both increased (38, 41, 49) and decreased (61) have been reported.

### 2.4 Lipid metabolism

#### 2.4.1 Palmitic acid

Palmitic acid is the most abundant saturated fatty acid in the human body, accounting for approximately 25% of the total fatty acids and 65% of the total saturated fatty acids in the human body (110). Interestingly, tumor cells can directly obtain fatty acids produced by adipocyte lysis in the TME, and an *in vitro* study proposed that adipocyte-derived palmitic acid promotes melanoma cell proliferation by activating protein kinase B (111, 112). Two studies found increased levels of palmitic acid in the blood of patients with NSCLC and pancreatic cancer and both proposed that metabolite panels containing palmitic acid could be used for the early diagnosis of diseases (19, 53). However, in another lung cancer study that included adenocarcinoma, squamous cell carcinoma, SCLC, and other pathological types, significantly lower palmitic acid levels were found in the case group than in the control group, and metabolic differences between SCLC and NSCLC may be responsible for this inconsistency with the aforementioned study (62).

#### 2.4.2 Linoleic acid

As an essential fatty acid, linoleic acid can only be acquired from food and cannot be synthesized because the body lacks relevant desaturation enzymes. In addition to participating in the composition of membrane phospholipids and cell energy supply, linoleic acid can be further converted to γ-linolenic acid and arachidonic acid through desaturation and extension reactions. Arachidonic acid derivatives such as prostaglandins and leukotrienes are important mediators involved in the inflammatory response and tumor development (113). Studies have identified significantly lower blood linoleic acid levels in patients with esophageal cancer (31), gastric cancer (63), and colon cancer (44), suggesting a key role of linoleic acid metabolism in gastrointestinal tumors.

#### 2.4.3 Phosphatidylcholines (PCs) and phosphatidylethanolamines (PEs)

PCs and PEs are the most abundant phospholipids in biofilms, and the ratio of PEs to PCs is critical in regulating lipoprotein secretion and maintaining mitochondrial function. In one MS study, lung cancer patients had lower levels of PCs and PEs in pleural effusions than did tuberculosis patients (66). Differences in blood PCs and PEs between cancer patients and controls were also reported in four studies (28, 64, 65, 70). Interestingly, in α-fetoprotein (AFP)-negative hepatocellular carcinoma, the concentrations of PC (22:6/18:2) and PC (18:2/18:2) were increased while the concentrations of PC (16:0/16:0) were decreased, which indicated that serological alterations in PCs would help us to identify patients with hepatocellular carcinoma missed by AFP at an early stage (64). Lysophosphatidylcholines (LPCs) and lysophosphatidylethanolamines are metabolic intermediates of PCs and PEs that are hydrolyzed by phospholipases. A significant body of literature has documented that changes in their levels in biological fluids are associated with early tumor diagnosis (35, 54, 59, 63, 67–70).

#### 2.4.4 Sphingomyelins (SMs) and ceramides (CERs) 

SMs and CERs are not only involved in maintaining the fluidity of biological membranes, but also act as important signaling molecules to regulate different life activities. SMs mainly promote cell proliferation and migration, while CERs can induce cell apoptosis and senescence (114). Five recent studies based on blood metabolomics observed differences in SMs between cancer patients and controls, four of which observed elevated levels of SMs in the patients with cancer (47, 70–72). One study showed reduced SM 42:2 and SM42:3 in patients with laryngeal cancer compared to patients with benign laryngeal tumors and healthy volunteers (73). A prospective study of 252 cases and 252 matched controls compared the differences in circulating lipid metabolites between groups, followed all enrolled subjects for up to 23 years, and concluded that: increased SMs and CERs predicted higher ovarian cancer risks (71).

#### 2.4.5 Glycocholic acid (GCA)

Bile acids are important products of cholesterol metabolism in hepatocytes and enter the intestine via the bile duct bile salt pump, thus contributing to lipids digestion and absorption (115). Farnesoid X receptor, the receptor of bile acids, plays a key role in maintaining bile acid balance in the liver and intestine (116). In the TME, inflammation inhibits the expression of the bile duct bile salt pump and the farnesoid X receptor, thus disrupting bile acid homeostasis and inducing an inflammatory response, ultimately creating a vicious cycle (117). GCA is the most abundant bile acid in the human body, whose changes in cancer have demonstrated its diagnostic potential. Two multicenter studies found higher levels of GCA in patients with hepatocellular carcinoma and colon cancer (57, 74). Another study proposed that the panel composed of five metabolites (creatine, inosine, β-sitosterol, sphinganine and GCA) had better diagnostic performance than carbohydrate antigen 125 and CEA (60).

#### 2.4.6 Estrogens and estrogen metabolites

Estrogens, a class of cholesterol derivatives, have been reported to be involved in the development of multiple malignancies, especially breast cancer (118, 119). In a randomized controlled trial, Dallal CM et al. found that postmenopausal women with elevated serum estradiol levels had a higher risk of breast cancer (75). Urinary estradiol levels in postmenopausal women have also been shown to be positively associated with the risk of breast cancer in two case-control studies (76, 77). Hydroxylation is the main pathway of estrogens metabolism. Based on the MS technique, studies have successively reported there is an inverse correlation between the ratio of serum 2-hydroxylation pathway metabolites to 16-hydroxylation pathway metabolites with breast cancer risk in postmenopausal women (75, 120, 121). However, due to the diversity of 16-hydroxylation pathway metabolites and the limited accuracy of specific estrogen metabolite concentrations determined by MS, the association between the ratio and breast cancer risk needs to be further verified (122). The formation of catechol estrogen quinones is a key molecular mechanism of estrogen carcinogenesis. It can further promote DNA mutation by reacting with DNA to form depurinating DNA-adducts (119). Previous studies have suggested that high-risk breast cancer patients have higher levels of depurinating DNA-adducts in circulating body fluids than healthy controls (123, 124), yet a recent study by Reding KW et al. found no correlation between urinary levels of depurinating DNA-adducts and breast cancer risk (125). In conclusion, these inconsistent findings indicate the necessity to further explore the reliability of circulating estrogens and their metabolites in predicting the risk of breast cancer.

### 2.5 Others

Hippuric acid, also known as “benzoylaminoacetic acid,” is a coupling of benzoic acid and glycine. Owing to its high stability, it was initially identified as a detoxification metabolite of toluene and is still used to predict occupational exposure to toluene (126). Moreover, hippuric acid is an intestinal metabolite of phenylalanine, and its role as a host-gut microbiome co-metabolite has received increasing attention (127). Although the mechanisms underlying the altered metabolism of hippuric acid in cancer development have not been elucidated, limited experimental studies have shown that a high-salt diet produces more hippuric acid by increasing the abundance of bifidobacteria in the mouse intestine, which enhances the tumor-killing function of natural killer cells (128). In three MS studies comparing the variance in urinary metabolites between patients with cancer and controls, reduced levels of hippuric acid were consistently observed, further suggesting its potential utility in cancer diagnosis (23, 78, 79).

Extracellular vesicles (EVs) serve as important mediators of cell-to-cell communication and offer new opportunities for identifying novel tumor markers (129). In addition to circulating proteins and nucleic acids, the role of EV-derived lipid metabolites in cancer diagnosis is also gaining attention (**Table 2**). Phospholipids are important components of extracellular vesicles, and there is growing evidence that PCs, PEs, and SMs in EVs derived from biological fluids show significant differences between cancer patients and non-cancer controls (130–132, 134–136, 139). It has been proposed that changes in the fatty acids content of blood-derived extracellular vesicles may indicate the presence of cancer (133, 136–138, 140). Although there is currently no consensus on the diagnostic value of extracellular vesicle-derived lipid metabolites in different tumors, these findings suggest a great potential for metabolomics-based extracellular vesicle assays to advance into clinical practice.

**Table 2:** Summary of extracellular vesicles lipidomics biomarkers for cancer diagnosis.

Disease	Sample	Controls	Lipids and derivatives	Refs
Lung cancer	Serum	Benign lung nodules and healthy donors	CE(19:2), CER(42:1), TG(54:2), PC(38:6), PC(38:7), PC(37:5), PC(38:5), TG(56:8)	(130)
	Pleural effusion	Tuberculosis	PC (35:0), SM (44:3)	(131)
Breast cancer	Plasma	Healthy donors	PC ae C40:6, LPC a C26:0, PC aa C38:5, PC ae C40:2, PC ae C34:2, PC ae C32:2, PC ae C38:3, SM (OH) C16:1	(132)
Prostate cancer	Plasma	Tumor-free controls	Hydroxyoctanoic acid	(133)
	Urine	Benign prostate hyperplasia	PCs, FA esters, sterols	(134)
		Healthy donors	Lactosylceramide (d18:1/16:0), PS 18:1/18:1,	(135)
Colorectal cancer	Serum	Healthy donors	Phospholipids, FAs, sphingolipids	(136)
	Plasma	Healthy donors	FAs	(137)
Hepatocellular Carcinoma	Plasma	Cirrhosis	FAs, phospholipids, sulfatides	(138)
Malignant melanoma	Serum	Healthy donors	PC 16:0/0:0	(139)
			FAs	(140)

CE, cholesteryl ester; CER, ceramide; TG, triglyceride; PC, phosphatidylcholine; SM, sphingomyelin; LPC, lysophosphatidylcholine; FA, fatty acid; PS, phosphatidylserine.

## 3 Metabolomic biomarkers for distinguishing type, grade and stage of cancer

In recent years, metabolic differences in different types of cancer have also been noted. Studies have not only identified metabolites that help to detect specific pathological types but also proposed potential metabolic markers to distinguish between the presence or absence of genetic mutations and primary or secondary tumors. In addition, the non-invasive and convenient characteristics of blood and urinary metabolomics make it a useful tool for predicting cancer grade and stage, which may instruct clinicians to develop appropriate treatment plans for patients (**Table 3**).

**Table 3:** Summary of biofluid metabolomic biomarkers for typing, grading and staging in cancer.

Application	Disease	Sample	Subjects	Metabolites	Refs
Typing	Lung cancer	Plasma/serum	Squamous cell carcinoma Adenocarcinoma SCLC	Palmitic acid, heptadecanoic acid, pentadecanoic acid, acylcarnitine C8:1, ornithine	(62)
			Squamous cell carcinoma Adenocarcinoma	Kynurenine-tryptophan ratio	(141)
			Primary lung cancer Pulmonary metastatic carcinoma	L-octanoylcarnitine, retinol, decanoylcarnitine	(142)
		Pleural effusion	Lung cancer with EGFR mutation Lung cancer without EGFR mutation	Butanoic acid, (tetrahydro-2-furanyl) methyl ester, 1-hexene, 3,4-dimethyl-, 2-undecen-4-ol (Receiver operating characteristic analysis) 2-undecen-4-ol, 2H-tetrazole, 2-methyl-, 2-propanol, 1-chloro-3-propoxy-,4H-1,2,4-Triazol-4-amine, cyclobutylamine, butanoic acid, (tetrahydro-2-furanyl) methyl ester hexane, 2,3,5-trimethyl-,1H-Tetrazole-1-ethanol, cyclopropene, allyl acetate (Bootstrapped t-test)	(143)
	Brain tumors	Cerebrospinal fluid	PCNSL SCNSL MBT	Inositol phosphate, homocysteine, valyl-methionine, 5-aminoimidazole (PCNSL versus SCNSL) Butyrylcarnitine, 3-dehydrocarnitine,2-furoylglycine, hypotaurine, L-glutamic, N-butyrylglycine, pyruvic acid, citric acid, phytosphingosine etc. (PCNSL versus MBT) 1-methyladenosine, citric acid, valyl-methionine, L-glutamic, hypotaurine (SCNSL versus. MBT)	(144)
	Ovarian cancer	Serum	Serous cystadenocarcinoma Non-serous serous carcinoma	Phosphatidic acid (38:4), sphingosine-1-phosphate (t16:1), N-arachidonoyl taurine (C20:4), aldosterone, lysophosphatidylinositol(O-32:1),18-hydroxycorticosterone, stearidonic acid, tetradecanoylcarnitine, choladien-24-oic acid, hydroxy-5-cholenoic acid, monoacylglycerol (18:2), kynurenine	(145)
Grading	Bladder cancer	Serum	High grade Low grade	Leucine, histidine, alanine, 3-methyl-2-oxovalerate, tyrosine	(146)
	Meningioma	Plasma	High grade Low grade	Arginyl-Proline, PS (44:6), 3-O-sulfogalactosylceramide (42:2), PS (36:5), CER (40:1)	(147)
Staging	Lung cancer	Plasma	Early stage (stage I/II) Advanced stage (stage III/IV)	Palmitic acid, heptadecanoic acid, ornithine, tridecanoic acid, stearic acid	(62)
	Non-small cell lung cancer	Plasma	Early stage (stage I/II/III) Advanced stage (stage IV)	D-phenylalanine, phenylacetic acid, o-phosphoethanolamine, dehydroepiandrosterone, uric acid, α-D-glucose, D-4-hydroxy-2-oxoglutarate, xanthosine, allocholic acid, 5-aminopentanoic acid, D-fructose	(148)
	Esophageal squamous cell carcinoma	Plasma/serum	Stage Tis Stage I-II Stage III-IV	Indoleacrylic acid, LPC (20:5), LPE (20:4)	(59)
	Esophageal squamous cell carcinoma	Plasma/serum	Stage I–IIA Stage IIB–IV	Hyodeoxycholic acid, (2S,3S)-3, methylphenylalanine, carnitine C9:1, indole-3-carboxylic acid	(149)
	Colorectal cancer	Plasma/serum	Stage I–II Stage III–IV	Glycerophospholipids, SM C18:0, citrullin	(150)
			Advanced stage (stage III/IV)	Hydroquinone, leucenol and sphingomyelin	(151)
	Bladder cancer	Serum	Stage Ta/T1 Stage T2	Histidine, alanine, tryptophan, glutamine, glycine, methylhistidine, choline, isobutyrate, threonine	(146)
	Renal cell carcinoma	Urine	Stage I–II Stage III–IV	l-kynurenine, l-glutamine, fructose 6-phosphate, butyrylcarnitine	(22)
	Prostate cancer	Serum and urine	Advanced stage (stage T2/T3)	Citric acid	(152)

EGFR, epithelial growth factor receptor; SCLC, small cell lung cancer, epithelial growth factor receptor; PCNSL, primary central nervous system lymphoma; SCNSL, secondary central nervous system involvement of systemic lymphoma; MBT, lung adenocarcinoma with brain metastases; PS, phosphatidylserine; CER, ceramide; LPC, lysophosphatidylcholine; LPE, lysophosphatidylethanolamine; Tis, tumor in sit; SM, sphingomyelin.

### 3.1 Type

#### 3.1.1 Glucose metabolism

Both primary brain tumors and secondary brain metastases are important reasons for short survival and high mortality rates. Metabolites of brain tumors can be released directly into the cerebrospinal fluid; therefore, abnormal metabolic variations in cerebrospinal fluid can effectively reflect the pathophysiological changes of brain tumors. One cerebrospinal fluid metabolomics study described the metabolic characteristics of primary central nervous system lymphoma, secondary central nervous system involvement of systemic lymphoma (SCNSL), and lung adenocarcinoma with brain metastases (MBT) by liquid chromatography-quadrupole time-of-fight spectrometric and cross−contrasted the differential metabolites of patients with these three types of brain tumor (144). Compared with MBT, SCNSL had lower levels of pyruvate, a common substrate for aerobic and anaerobic oxidation. Additionally, there was a higher level of citric acid in MBT than in primary central nervous system lymphoma and SCNSL, implying a more active aerobic oxidative metabolism in MBT. Epithelial growth factor receptor (EGFR) mutations are common in lung cancer, and the identification of gene mutation types is becoming increasingly crucial because of the clinical efficacy of the latest generation of EGFR-targeted drugs. Chen et al. used GC-MS to compare the volatile metabolites of malignant pleural effusion in lung cancer patients with or without EGFR mutations (143). There was a clear separation between the clustering of patients with and without EGFR mutations from the score plot. After performing ROC analysis and bootstrapped t-test, they identified 5 and 14 volatile organic compounds related to glucose synthesis and catabolism, respectively, for the early identification of EGFR mutation.

#### 3.1.2 Amino acid metabolism

Due to the presence of heterogeneity, significant amino acid alterations have also been observed in the biological fluids of patients with different pathological types of cancer. A study using MS analyzed the plasma metabolite differences in patients with three types of lung cancer: lung adenocarcinoma, lung squamous cell carcinoma, and SCLC (62). Ornithine has emerged as a possible biomarker, following the application of orthogonal projections to latent structures discriminant analysis (OPLS-DA). Based on previous studies (153), to further explore metabolic changes of kynurenine and tryptophan in different types of lung cancer, Mandarano et al. observed that patients with squamous cell carcinoma have a higher serum kynurenine/tryptophan ratio than patients with lung adenocarcinoma (141). Kynurenine was also highlighted in another MS study on epithelial ovarian cancer, which explored serum metabolomic alterations in four different histopathological types (145). A panel of 12 metabolites containing kynurenine were identified as potential markers for distinguishing serous carcinoma from non-serous carcinoma by ROC analysis.

#### 3.1.3 Lipid metabolism

Changes in lipid metabolites have been associated with the origin of malignant lung nodules. In a study conducted by Liu et al. involving 16 patients with pulmonary metastatic carcinoma and 80 patients with primary lung carcinoma, a group of lipid metabolic biomarkers consisting of L-octanoylcarnitine, retinol, and decanoylcarnitine were able to predict metastatic lung cancer (AUC > 0.95) (142). Abundant differential metabolic molecules associated with lipid metabolism between serous ovarian carcinoma and non-serous ovarian carcinoma have also been identified by Olkowicz et al., such as phosphatidic acid (38:4), monoacylglycerol (18:2), stearidonic acid, choladien-24-oic-acid and hydroxy-5-cholenoic acid (145).

### 3.2 Grade

One reported NMR spectroscopic metabolic panel could distinguish patients with low-grade bladder cancer (n = 54) from those with high-grade bladder cancer (n = 41) through a permutation analysis-validated OPLS-DA model comprising five metabolites (leucine, histidine, alanine, 3-methyl-2-oxovalerate, and tyrosine) (146). In addition to bladder cancer, an OPLS-DA score plot by Kurokawa et al. showed patients with meningioma were clustered according to different grades (147). Finally, Arginyl-Proline, PS (44:6), 3-O-sulfogalactosylceramide (42:2), PS (36:5) and CER (40:1) were identified as serum metabolic markers for high-grade meningiomas. Arginyl-Proline is a dipeptide formed by the dehydration condensation of arginine and proline, the increase in which indicates that high-grade meningiomas have a greater capacity for proliferation. The four remaining metabolic markers indicated the significant involvement of phospholipid metabolism in the pathological progression of high-grade meningiomas.

### 3.3 Stage

#### 3.3.1 Glucose metabolism

Attempts to use metabolomic techniques identifying biomarker for advanced renal cell carcinoma have been reported. Sato et al. reported that a panel of four metabolites, l-kynurenine, l-glutamine, fructose 6-phosphate, and butyrylcarnitine, could predict stage III/IV disease with high accuracy (sensitivity 88.5% and specificity 75.4%) (22). Higher levels of glucose-6-phosphate may indicate an increase in the energy requirements obtained through glycolysis. GLUT5, a specific fructose transporter, has recently been reported to interact with interleukin-6 to participate in tumor progression by promoting fructose uptake (154). In an MS study of NSCLC patients, 11 metabolites including D-fructose were identified that differed markedly in plasma between patients in the early and advanced stages according to partial least squares discriminant analysis (variable importance in projection ≥ 1) and Mann-Whitney test (P-value ≤ 0.05) (148). Citric acid, an intermediate product of the TCA cycle, is crucial for the interactions between glucose and lipid metabolism. In a study on prostate cancer, Buszewska-Forajta et al. observed low levels of citric acid in the blood, urine, or tissue of patients in the advanced stages, suggesting that citric acid and prostate cancer progression are closely linked (152).

#### 3.3.2 Amino acid metabolism

In an NMR study, a metabolite panel containing seven amino acids demonstrated an AUC > 0.80 in distinguishing between stage Ta/T1 and T2 bladder cancer, and all of these amino acids were relatively reduced in stage T2 (146). Apart from D-fructose, alterations in plasma levels of intermediates in the phenylalanine metabolism pathway, such as D-phenylalanine and phenylacetic acid, have also been observed to differ in patients with early- and advanced- stage lung cancer (148). (2S,3S)-3-Methylphenylalanine, another phenylalanine derivative, was identified as a putative biomarker of esophageal squamous cell carcinoma progression using least absolute shrinkage and selection operator regularization and random forest (149).

#### 3.3.3 Lipid metabolism

In the absence of inexpensive and effective markers, colonoscopy and computed tomography remain the primary methods of clinically staging of colon cancer. A combination of tandem MS and logistic regression analysis of serum samples collected from 744 patients with colon cancer generated metabolomic profiles that distinguished stages III-IV from stages I-II (150). The top ten metabolites included nine PCs and SM (C18:0), indicating that phospholipid metabolism was disturbed during colorectal cancer progression. Hydroquinone and sphingomyelin, two other molecules of the lipid metabolism pathway, were identified by Rao et al. as new markers for the early detection of advanced colorectal cancer and showed a good correlation with the traditional markers CA199 and CEA (151). However, owing to the small sample size and lack of early-stage patients for comparison, further verification is required. In an untargeted plasma metabolomics study, LPC (20:5) and LPE (20:4) were identified as putative biomarkers of esophageal squamous cell carcinoma progression (59), which may be related to their involvement in pathological injury to the cell membrane. After successfully identifying plasma metabolites that help in the diagnosis of lung cancer through metabolomics, Qi et al. attempted to identify metabolic markers that could distinguish different stages of lung cancer (62). In their study, an optimized discriminant logistic regression model generated by least absolute shrinkage and selection operator regularization of five variable importance in projection-selected metabolites (palmitic acid, heptadecanoic acid, ornithine, tridecanoic acid and stearic acid) was able to effectively discriminate between early and advanced stages. Four are fatty acids, except for ornithine, implying that fatty acid biosynthesis is required for tumor growth and proliferation.

## 4 Metabolomic biomarkers for monitoring cancer treatment efficacy and prognosis

Most studies have identified metabolomic markers for predicting therapy efficacy and prognosis in blood, urine, and other biofluids from patients with cancer (**Table 4**), which may help clinicians screen good responders and prevent delaying the malignant condition in poor responders by adjusting their treatment strategies timely.

**Table 4:** Summary of biofluid metabolomic biomarkers for treatment response and prognosis prediction in cancer.

Disease	Sample	Metabolites	Potential clinical uses	Refs
Lung cancer	Serum/plasma	SM 42:2, SM 35:1, PC (16:0/14:0), PC (14:0/16:1), SM 38:3, CER (d18:1/24:1)	Recurrence after surgery	(35)
		3-hydroxyanthranilic acid	Efficacy of anti-PD-1 immunotherapy	(43)
		Serine, glycine, arginine, quinoline acid	Efficacy of anti-PD-1 immunotherapy	(155)
Esophageal squamous cell carcinoma	Serum/plasma	Isocitric acid, linoleic acid, citric acid, L-histidine, 3’4dihydroxyhydrocinnamic acid	Efficacy of neoadjuvant chemoradiotherapy	(156)
		Kynurenine, 2-piperidinone, hippuric acid, LPC (14:0)	Survival	(157)
		Monoglyceride (20:4) isomer, 9,12-octadecadienoic acid, L-isoleucine	Survival	(149)
Pancreatic cancer	Serum/plasma	Succinic acid, gluconic acid,	Metastasis	(60)
		LPC18:2, CER 36:1, CER 38:1, CER 42:2, PC 32:0, PC O-38:5, SM 42:2	Survival	(67)
	Urine	Trigonelline, hippurate, myoinositol	Survival	(158)
Hepatocellular carcinoma	Serum	Formate	Microvascular invasion	(159)
		Retinol, retinal	Survival	(160)
Gastric cancer	Peritoneal lavage fluid	Glyceraldehyde-3-phosphate sulfite	Peritoneal metastasis	(161)
Colorectal cancer	Serum	Diacron’s reactive oxygen metabolites, total thiol	Survival	(162)
Renal cell carcinoma	Urine	Lactic acid, glycine, succinic acid, 2-hydroxyglutarate, kynurenic acid	Recurrence after surgery	(163)
Bladder cancer	Serum	Dimethylamine, malonate, lactate, glutamine, histidine, valine	Postoperative dynamic monitoring	(164)
		Taurine, glutamine, glycine, hypoxanthine	Efficacy of neoadjuvant chemotherapy	(165)
Ovarian cancer	Plasma	Kynurenine/tryptophan ratio	Survival	(166)
	Serum	Hydroxybutyric acid, maleic acid, D-dysteine, N-acetylasparagine, 3-hydroxy-2-methylpyridine-4,5-dicarboxylate, dihydroneopterinphosphate	Efficacy of chemotherapy	(167)
Prostate cancer	Serum	1-methyladenosine, phosphatidic acid 18:0-22:0	Recurrence	(168)
Osteosarcoma	Serum	5-aminopentanamide, FA 18:3 + 2O	Lung metastasis	(21)
Diffuse Large B-Cell Lymphoma	Serum/plasma	Malate	Survival	(17)
		Valine, hexadecenoic acid, pyroglutamic acid	Survival	(169)
Multiple myeloma	Serum	Cysteine, hypotaurine	Efficacy of chemotherapy	(170)
		Ethanoic acid, xylitol	Survival	

SM, sphingomyelin; PC, phosphatidylcholine; CER, ceramide; PD-1, programmed cell death protein 1; LPC, lysophosphatidylcholine; FA, fatty acid.

### 4.1 Glucose metabolism

Blood metabolites collected before treatments have predictive power for therapeutic response and survival of patients with cancer. One study reported lower levels of isocitric acid and citric acid in the non-pathological complete response group than in the pathological complete response group, suggesting that these two compounds may predict patient responses to neoadjuvant chemoradiotherapy (156). Using multivariate logistic regression analysis, another MS study determined a five-metabolite panel containing lactic acid, 2-hydroxyglutaric acid, and succinic acid that could predict the recurrence of renal cell carcinoma after surgery with a sensitivity and specificity of 88.9% and 88.0%, respectively (163). Microvascular invasion is a detrimental factor affecting the surgical outcome and prognosis of patients with hepatocellular carcinoma, sensitive detection methods are still lacking because of its insidious nature. Lee et al. presented an OPLS-DA model in their NMR study that effectively differentiated between patients with and without microvascular invasion (159). Combining formate and CA199 improved the ability to predict microvascular invasion, which was confirmed in the validation cohort. Differential metabolites have also been detected in the peritoneal lavage fluids of gastric cancer patients and those with peritoneal metastasis tended to have higher glyceraldehyde-3-phosphate (161).

### 4.2 Amino acid metabolism

Plasma metabolomics measured with high-performance liquid chromatography for NSCLC patients receiving anti-programmed cell death protein 1 therapy with good survival have also been compared with those obtained from patients with poor survival and could be successfully separated in a multivariate model (concordance index = 0.775, hazard ratio = 3.23) (155). Quinolinic acid, a tryptophan intermediate metabolite mentioned in this study, was negatively correlated with the immunotherapy efficacy in NSCLC patients. Another tryptophan catabolite, 3-hydroxyanthranilic acid, was also proposed as a potential indicator of adverse clinical outcomes in NSCLC patients receiving anti-programmed cell death protein 1 therapy (43). In addition to NSCLC, abnormal tryptophan catabolism is also found in esophageal and ovarian cancers. Plasma kynurenine levels have been proposed to be negatively correlated with patient survival (157, 166). According to serum metabolomic profiles detected by NMR, the levels of three amino acids (glutamine, histidine and valine) in bladder cancer patients decreased markedly after surgery and approached those in healthy controls (164). In an untargeted study using both NMR and MS, significant increases in glycine and decreases in taurine and glutamine levels were observed in patients resistant to neoadjuvant chemotherapy (165). Monitoring changes in blood amino acids has also been reported to effectively predict chemotherapy efficacy and survival in patients with hematological malignancies (169, 170).

### 4.3 Nucleotide metabolism

Recent studies have also revealed that metabolites of nucleotide metabolic pathways can be potential predictors of cancer prognosis. Jung et al. divided 18 patients with bladder cancer who received neoadjuvant chemotherapy into sensitive (n = 6) and resistant (n = 12) groups based on their therapeutic response and observed higher serum hypoxanthine levels in the resistant group (P-value < 0.05) (165). Furthermore, elevated serum levels of 1-methyladenosine (a purine nucleotide metabolite) are associated with an increased risk of prostate cancer recurrence (168).

### 4.4 Lipid metabolism

Surgery remains the preferred treatment for resectable lung cancer. Yang et al. proposed a panel composed of six phospholipids (SM 42:2, SM 35:1, SM 38:3, PC 30:0, PC 30:1and CER 42:2) through the long-term follow-up of patients undergoing surgery, which could effectively distinguish between patients with postoperative recurrence and non-recurrence (35). Pancreatic cancer and liver cancer are highly malignant and rapidly evolving diseases for which convenient and sensitive clinical prognostic markers are still lacking. Recent researches have shown that lipids and lipid molecules in the blood are strongly related to patient survival. By establishing proportional hazards model, Wolrab et al. found LPC 18:2 and PC O-38:5 exhibited a positive and negative correlation with the overall survival of patients with pancreatic cancer, respectively (67). Similarly, liver cancer patients with low levels of retinol and retinal showed poor survival, so these two metabolites were identified as new prognostic markers superior to AFP (160).

### 4.5 Others

Based on the role of oxidative stress in the pathological progression of colon cancer, a large-scale study enrolled 3361 patients with colon cancer and followed them for up to 6 years to explore the correlation between oxidative stress products and patient prognosis (162). A higher pre-treatment serum abundance of total thiol and the total thiol to diacron reactive oxygen metabolites ratio were associated with lower mortality, particularly in stage IV patients. In a pancreatic cancer study, a panel of three metabolites (trigonelline, hippurate and myoinositol) measured by 1H-labeling NMR had the potential to stratify postoperative patients into good or poor outcomes (158).

## 5 Major analytical techniques in metabolomics

NMR is a technique that utilizes the resonance phenomenon of nuclei in a magnetic field to measure the resonance frequency and intensity of different nuclei. It has several advantages, including fast analysis, non-destructive testing, and good repeatability due to the simple sample pretreatment process. However, NMR also presents the limitations of poor sensitivity and resolution when characterizing metabolites that are present at low concentrations, are unknown, or have overlapping spectra (171). The development and application of two-dimensional (2D) NMR technology (172) and cryogenic probe 13C NMR spectroscopy (173) are the main means to improve resolution and enhance sensitivity. Mass Spectrometry (MS) is a technique that ionizes molecules by bombarding them with electrons. The ions are then separated based on their mass-to-charge ratio, and the relative peak intensities of different ions are measured (174). Higher sensitivity is the main advantage of MS compared to NMR. GC-MS and LC-MS have different applications for metabolomics studies depending on the nature of the isolated metabolites (175). LC-MS is primarily used to analyze non-volatile and unstable substances, while GC-MS is suitable for the analysis of volatile metabolites. The advent of UPLC-MS/MS technology has further improved the analytical speed and sensitivity of MS technology. In addition to separation-based MS technology, nanoparticle-enhanced laserdesorption/ionization-mass spectrometry (NPELDI-MS) has significantly advanced metabolomics due to its increased sensitivity, speed, and mass accuracy (176). A large-scale gastric cancer study combined NPELDI-MS technology with machine learning algorithms to construct a blood metabolic marker panel for the early detection of gastric cancer that significantly outperforms traditional tumor markers (176). **Table 5** compared the characteristics of NMR and MS techniques.

**Table 5:** Comparisons of common analytical techniques in metabolomics.

	NMR	MS
Sample preparation	Simple and non-destructive	Complex and destructive
Sensitivity	Low	High
Resolution	Low	High
Repeatability	High	Low
Quantitative capability	Absolute quantification	Relative quantification
Qualitative capability	Accurate but limited databases	LC-MS: Relatively difficult and limited databases GC-MS: Relatively easy and robust databases

NMR, nuclear magnetic resonance; MS, mass spectrometry; LC-MS, liquid chromatography-mass spectrometry; GC-MS, gas chromatography-mass spectrometry.

## 6 Limitations and challenges of cancer metabolomic biomarkers in liquid biopsy

By offering a comprehensive “fingerprint” of metabolite alterations across various biofluids, metabolomics has presented an array of potential biomarkers in cancer management. However, multiple studies have reported inconsonant changes in metabolites for a specific type of cancer. It is important to fully understand the current limitations in metabolomics research and find solutions to these challenges in future studies.

The heterogeneity of the study population is an important reason for the differences between studies. Metabolic information carried by body fluids not only comes from the internal metabolism of the entire body but also is affected by the external environment. Therefore, a patient’s metabolic profile can be affected by various factors such as age, gender, dietary habits, medications, and comorbidities, it is necessary to set up strict exclusion and control matching criteria to minimize the impact of confounding factors. Moreover, considering the existence of individual heterogeneity, expanding the sample size and establishing a validation cohort are key to improving the objectivity of the research findings.

Differences in sample selection and preparation significantly contribute to the variation in results. Various biological fluids, including blood, urine, pleural fluid, peritoneal fluid, and cerebrospinal fluid, can be analyzed for metabolomics. Peripheral blood and urine are the first choice for most metabolomics studies because of their high clinical accessibility. Selecting appropriate sample sources based on the physiological characteristics of tumors can provide more valuable information for metabolomics studies. For example, as a crucial component of the lung cancer microenvironment, pleural effusion can more directly reflect metabolic changes in lung cancer patients and is less influenced by the body’s systemic metabolism (143). Uniform collection time and appropriate storage conditions are crucial for accurate metabolomics data. Therefore, proper sample preparation is vital and should align with the research design and sample requirements (177).

Disagreements among different studies may be related to variations in analytical platforms and statistical methods. Most metabolomics studies rely on either MS or NMR techniques for metabolite detection and analysis. The lack of uniform criteria for selecting differential metabolites and the use of a single analytical platform have led to unsatisfactory comparisons between studies. Since NMR and MS technologies are different in principles and strengths, combining these two technologies is a crucial approach to fully harnessing the potential of metabolomics in cancer research (178). The selection of suitable statistical methods is also a crucial step in identifying metabolic markers specific to tumors. The multivariate analysis approach enables researchers to gain a comprehensive understanding of the contribution of various metabolites in cancer development and facilitates the establishment of more reliable metabolic models. Besides commonly used multivariate analysis methods such as Principal Component Analysis and OPLS-DA, multivariate logistic regression models that include clinicopathologic factors and contributing metabolites are a recommended option (179). Furthermore, the use of machine learning algorithms has facilitated the efficient analysis of complex metabolomics datasets (167). However, selecting appropriate algorithms and improving their generalizability remain issues for future consideration (180).

## 7 Conclusions and prospects

**Figure 2:**
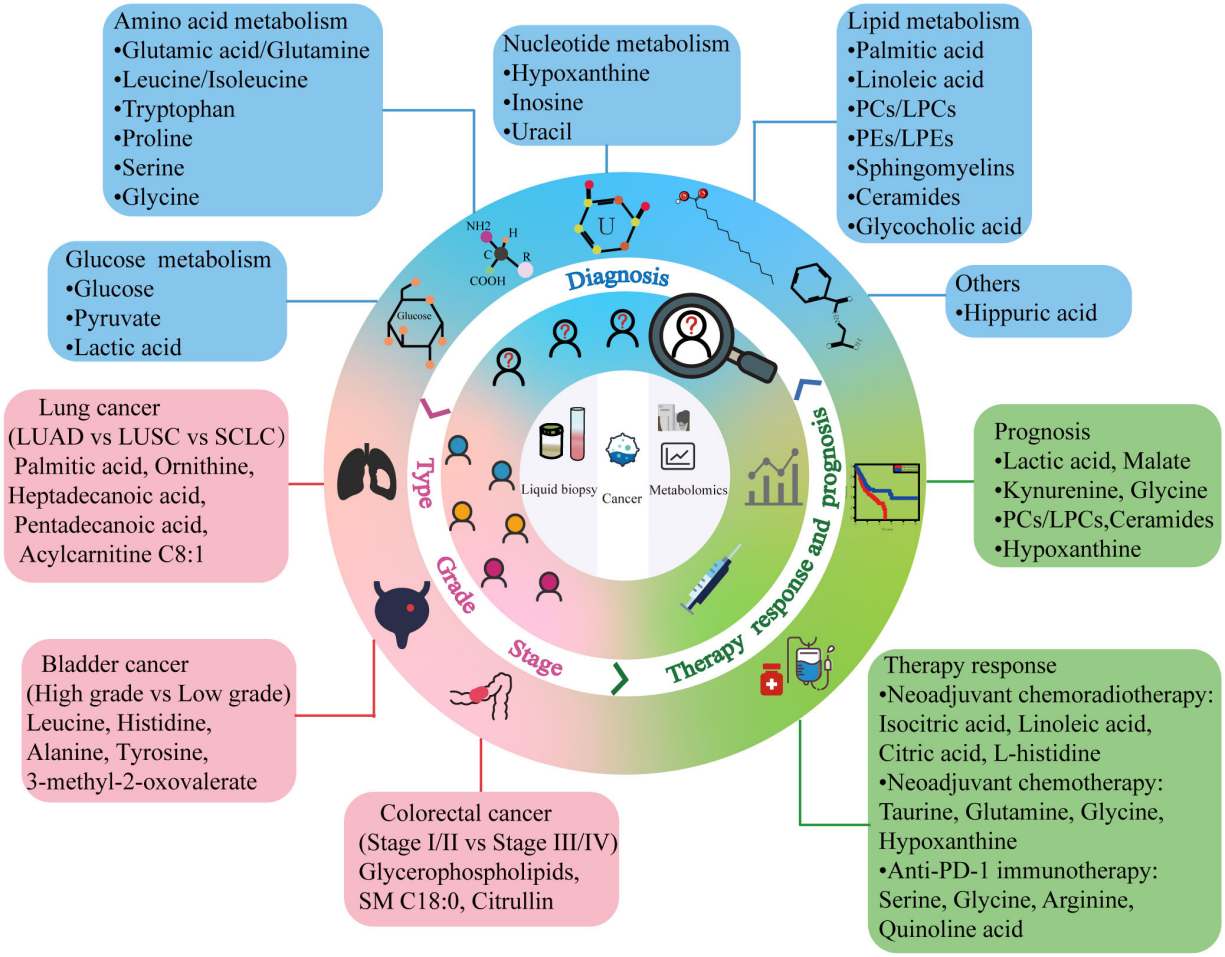
Cancer metabolic markers obtained by liquid biopsy. Combining liquid biopsy with metabolomics can provide potential biomarkers for different aspects of clinical cancer management. PC, phosphatidylcholine; LPC, lysophosphatidylcholine; PE, phosphatidylethanolamine; LPE, lysophosphatidylethanolamine; LUAD, lung adenocarcinoma; LUSC, lung squamous cell carcinoma; SCLC, small cell lung cancer; PD-1, programmed cell death protein 1.

Advances in liquid biopsy are breaking new grounds for cancer diagnosis and treatment. Although liquid biopsy is not yet a complete substitute for tissue biopsy, it is a convenient, sensitive, and safe strategy for future clinical cancer management. Considering the intricate roles of metabolites and their related pathways in cancer development, metabolomic biomarkers identified in recent biofluid metabolomics studies would offer valuable information for tumor diagnosis, classification, and prognosis prediction (**Figure 2**). Metabolites that differ significantly in the body fluids of patients with cancer compared to those with benign disease and healthy individuals can aid clinicians in recognizing the presence of tumors early, particularly in patients who test negative for traditional tumor markers (181). Identifying the differential metabolites that vary in distinct tumor types, grades, and stages assists in understanding the pathological characteristics and malignancy of tumors and provides clues for appropriate treatment plans. Tracking metabolite changes before and after treatment enables real-time monitoring of patient responses and timely identification of patients with a favorable prognosis. However, most of the current findings in this field are limited to single-center, small-sample studies. Inter-agency collaboration or the promotion of centralized testing centers should be encouraged to ensure accurate identification of metabolomic biomarkers. Only through validation in multi-center studies and clinical trials can these metabolic markers advance to the clinical translation stage. Therefore, caution must be exercised at this time when considering the practical application of these results in the clinic. Notably, differential metabolites, especially those overlapping metabolites between studies, can guide researchers to further explore metabolic mechanisms in cancer development and provide a theoretical basis for the identification of metabolic markers. Furthermore, recent studies have shown that combining metabolomics with other omics can result in more sensitive cancer marker panels for detecting changes in a patient’s condition (182, 183). The biomolecular mechanisms underlying metabolic markers may make targeting metabolism for antitumor therapy a promising option in clinical settings.

## Author contributions

WW: Writing – original draft. SZ: Writing – original draft. YP: Writing – review & editing. LW: Conceptualization, Supervision, Writing – review & editing. YZ: Conceptualization, Funding acquisition, Supervision, Writing – review & editing.
